# Associations of vitamin D status with all-cause and cause-specific mortality in long-term prescription opioid users

**DOI:** 10.3389/fnut.2024.1422084

**Published:** 2024-06-18

**Authors:** Shan Dai, Junpeng Wu, Peng Wang, Zhenhua Hu

**Affiliations:** ^1^Department of Anesthesiology and Perioperative Medicine, People’s Hospital of Zhengzhou University, Henan Provincial People’s Hospital, Zhengzhou, Henan, China; ^2^Department of Anesthesiology, Key Laboratory of Precision Anesthesia and Perioperative Organ Protection of Guangdong Province, Nanfang Hospital, Southern Medical University, Guangzhou, Guangdong, China

**Keywords:** prescription opioids, pain, mortality, NHANES, 25-hydroxyvitamin D

## Abstract

**Objective:**

This study aimed to investigate the association between serum 25-hydroxyvitamin D (25(OH)D) concentrations and mortality in long-term prescription opioid users.

**Methods:**

The study included 1856 long-term prescription opioid users from the National Health and Nutrition Examination Survey (NHANES, 2001–2018). Mortality status were determined by matching with the National Death Index (NDI) records until December 31, 2019. Multivariable Cox proportional hazard models were constructed to assess the association.

**Results:**

Over a median follow-up period of 7.75 years, there were 443 cases of all-cause mortality, including 135 cardiovascular disease (CVD) deaths and 94 cancer deaths. After multivariable adjustment, participants with serum 25(OH)D concentrations within 50.00 to <75.00 nmol/L and ≥ 75 nmol/L had a lower risk of all-cause mortality, with hazard ratios (HRs) of 0.50 (95% confidence interval [CI] 0.29, 0.86) and 0.54 (95% CI 0.32, 0.90), respectively. Nevertheless, no significant association was found between serum 25(OH)D concentrations and the risk of CVD or cancer mortality. The RCS analysis revealed a non-linear association of serum 25(OH)D concentration with all-cause mortality (*p* for non-linear = 0.01). Per 1-unit increment in those with serum 25(OH)D concentrations <62.17 nmol/L corresponded to a 2% reduction in the risk of all-cause mortality (95% CI 0.97, 1.00), but not changed significantly when 25(OH)D concentrations ≥62.17 nmol/L.

**Conclusion:**

In conclusion, a non-linear association existed between serum 25(OH)D concentrations and all-cause mortality in long-term prescription opioid users. Maintaining serum 25(OH)D concentrations ≥62.17 nmol/L may be beneficial in preventing all-cause mortality in this population.

## 1 Introduction

Prescription opioids are widely recognized as the most commonly used and effective analgesics for managing moderate to severe pain, and the rates of prescription opioid use are increasing in many countries (1). Despite their significant pain-relieving properties, long-term use of prescription opioids increases the risk of overdose, misuse, addiction, and other associated risks (2), with 16,706 fatalities attributed to prescription opioid overdose in 2021 (3). Increasing evidence suggests long-term prescription opioid use is associated with all-cause mortality (4–6). However, the results regarding the association between long-term prescription opioid use and cardiovascular or cancer mortality were contradictory. Ekholm et al. (6) reported no association was observed for long-term prescription opioid use with cardiovascular and cancer mortality. In contrast, Nalini et al. (7) found long-term opiate use was associated with an increased risk of cardiovascular mortality, and Song et al. (4) also observed a higher mortality rate due to cancer or circulatory system diseases in long-term opioid users.

Vitamin D is a steroid hormone, and 25-hydroxyvitamin D (25(OH)D) serves as the predominant circulating form of vitamin D in the bloodstream. Vitamin D in the body is primarily synthesized through the skin upon exposure to ultraviolet radiation, and it can also be obtained in small quantities from the diet (8). Apart from its role in maintaining bone health, vitamin D plays a crucial role in immune regulation, cell growth, and cellular differentiation (9). Several epidemiological studies have identified a link between low 25(OH)D concentrations and an increased risk of mortality in the general population (10, 11). Nevertheless, recent intervention studies have failed to demonstrate the benefit of vitamin D supplementation on mortality (12–14). Prior study have reported that chronic pain patients with vitamin D deficiency faced an increased risk of receiving higher opioid doses and using them for extended periods (15). Recent study have indicated vitamin D deficiency was associated with an increased risk of prescription opioid use and the exacerbation of opioid addiction (16). The absence of vitamin D signaling can increase sensitivity to morphine reward, leading to greater exogenous opioid consumption (16). Furthermore, vitamin D may exert analgesic effects through stimulating the body’s anti-inflammatory response, scavenging reactive oxygen species, and regulating the endogenous opioid pathway, thereby affecting opioid use (17–19). Despite the presence of evidence indicates a connection between vitamin D deficiency and the use of prescription opioids, no reports have addressed the association of vitamin D deficiency with mortality in long-term prescription opioid users. Due to the variation in the impact of long-term prescription opioid use on mortality outcomes, further research is needed to elucidate the relationship between them.

To address this research gap, we conducted a prospective investigation to examine the associations of serum 25(OH)D concentrations with all-cause and cause-specific mortality in long-term prescription opioid users. We hypothesized that low 25(OH)D concentrations would be associated with an increased risk of all-cause and cause-specific mortality in this population.

## 2 Materials and methods

### 2.1 Study population

National Health and Nutrition Examination Survey (NHANES) is a nationally representative cross-sectional survey that utilizes a stratified multistage sampling design to evaluate the health and nutritional status of the non-institutionalized population in the United States (20). The research protocol was approved by the Institutional Review Board of the National Center for Health Statistics (NCHS), and written informed consent was obtained from all participants. Since the de-identified data analyzed during the current study were publicly available from NHANES, the study did not require any review board approval again.

This study included a total of 50,201 participants aged ≥20 years from 2001 to 2018. We excluded non-long-term prescription opioid users (*n* = 47,426), individuals receiving medications for opioid dependence or withdrawal (including buprenorphine; naloxone, buprenorphine; methadone, *n* = 79), those with missing vitamin D data (*n* = 331), those with missing follow-up data (*n* = 1), and those with missing covariate data (*n* = 508). Ultimately, the analysis included 1856 participants. The participant inclusion process was detailed in Supplementary Figure S1.

### 2.2 Measurement of serum 25(OH)D concentrations

Before 2005–2006, serum 25(OH)D concentrations were measured using the DiaSorin RIA kit (Stillwater, MN, USA). Since 2005–2006, a standardized liquid chromatography–tandem mass spectrometry (LC–MS/MS) method had been used to measure serum 25(OH)D concentrations. Following the analytical guidelines of Centers for Disease Control and Prevention (CDC), serum 25(OH)D concentrations from 2005 to 2006 and earlier were converted to equivalent concentrations from LC–MS/MS using regression, and LC–MS/MS equivalent data were utilized in all analyses.

### 2.3 Ascertainment of long-term prescription opioid users

During the household interview survey, participants were asked if they had taken a medication in the past month for which they needed a prescription. Participants who answered “yes” were asked to report the names of the medications through medication containers, verbal reports, or pharmacy receipts. Additionally, participants were also asked how long they had been taking the medication. All recorded medications were classified using the Multum Lexicon therapeutic classification system. Participants who reported the use of narcotic analgesics or narcotic analgesic combinations were considered as prescription opioid users, and the duration of prescription opioid use ≥90 days was considered as long-term use (21). Methadone, naloxone, and buprenorphine were often used to treat opioid dependence or withdrawal, we exclude participants taking these drugs from prescription opioid users.

### 2.4 Ascertainment of mortality

Mortality status and follow-up time were determined by matching with the National Death Index (NDI) records available until December 31, 2019. According to the International Classification of Diseases, 10th Revision (ICD-10), cardiovascular disease-specific mortality was defined as deaths due to heart disease (I00-I09, I11, I13, I20-I51) or cerebrovascular disease (I60-I6), while cancer-specific mortality was defined as deaths due to malignant neoplasms (C00-C97).

### 2.5 Covariates

Sociodemographic information [age, sex, race/ethnicity, education, poverty-income ratio (PIR)], living habits (physical activity, cotinine, alcohol consumption), comorbidity (history of hypertension, cardiovascular disease (CVD), diabetes, and cancer), as well as body mass index (BMI), were obtained from NHANES. Race/ethnicity was categorized as Non-Hispanic White, Non-Hispanic Black, Mexican American, and Other. Education was divided into less than high school, high school, and more than high school. PIR was classified as ≤130, 130–300%, and > 300% (22). Physical activity was calculated as weekly minutes of moderate and vigorous activity multiplied by metabolic equivalent (MET) levels. Alcohol consumption was categorized as never (<12 drinks in a lifetime), former (≥12 drinks in a year or ≥ 12 drinks in a lifetime but none in the past year), mild (female: <2 drinks/d, male: <3 drinks/d), moderate (female: 2 to <3 drinks/d, male: 3 to <4 drinks/d, or 2–4 binges/month), and heavy (female: ≥3 drinks/d, male: ≥4 drinks/d, or ≥ 5 binges/month) (23). BMI was calculated as weight (kilograms) divided by the square of height (meters) and classified as <25, 25–30, and ≥ 30 (24). Hypertension was assessed by systolic blood pressure ≥ 130 mmHg, diastolic blood pressure ≥ 80 mmHg, or taking antihypertensive drugs (25). Diabetes was determined by self-reported diagnosis by doctor or glycosylated hemoglobin (HbA1c) level ≥ 6.5% or taking anti-diabetic drugs. CVD was confirmed through self-reported diseases, including coronary heart disease, congestive heart failure, heart attack, stroke, and angina.

### 2.6 Statistical analyses

All analyses were conducted using appropriate sample weights to account for the complex sampling design of NHANES. Serum 25(OH)D concentrations were classified into four groups: severe deficiency (<25.00 nmoL/L), deficiency (25.00 to <50.00 nmoL/L), insufficiency (50.00 to <75.00 nmoL/L), and sufficiency (≥75.00 nmoL/L) (26). The reference group was defined as those with serum 25(OH)D concentrations <25.00 nmoL/L. Continuous variables and categorical variables were presented as means (standard errors, SE) and frequencies (weighted percentages), respectively. Differences between the four 25(OH)D groups were assessed using ANOVA for continuous variables and chi-square test for categorical variables. Multivariable Cox proportional hazards regression models were created to investigate the association between serum 25(OH)D concentrations and risks of all-cause or cause-specific mortality. Model 1 made no adjustments; model 2 was adjusted for age, sex, race/ethnicity; model 3 was further adjusted for education, alcohol consumption, cotinine, physical activity, BMI, PIR, hypertension, diabetes, cancer, and CVD. Restricted cubic spline analysis (RCS) with 5 knots (5th, 28th, 50th, 73th, and 95th) was applied to examine the non-linear relationship between serum 25(OH)D concentrations and all-cause mortality. Stratified analyses were further performed by age (<60 or ≥ 60 years), sex (male or female), race/ethnicity (Non-Hispanic White, Non-Hispanic Black, Mexican American, and Other), BMI (< 25, 25–30, and ≥ 30), and cancer (yes or no). We also conducted several sensitivity analyses: (1) Participants who died within 2 years of follow-up were excluded to minimize reverse causality; (2) Participants with a history of cardiovascular disease or cancer were further excluded; (3) Additionally, adjustments were made for the blood drawing season to account for the influence of seasonal variations on vitamin D status.

All analyses were conducted using R 4.2.3 (Boston, MA, USA), and a two-tailed *p*-value <0.05 was considered statistically significant.

## 3 Results

### 3.1 Characteristics of participants

Of the 1856 participants with long-term prescription opioid use (mean [SE] age, 54.12 (0.5) years; 808 male [weighted, 40.7%]; 1,060 [weighted, 78.5%] non-Hispanic White; mean [SE] serum 25(OH)D concentrations, 70.19 (1.2) nmol/l), 32.4% were with deficient vitamin D (<50 nmoL/L), and 67.2% were with insufficient vitamin D (<75.00 nmoL/L). Table 1 shows the baseline characteristics of the study participants stratified by serum 25(OH)D concentrations. Compared to sufficient vitamin D (≥75.00 nmoL/L), the age of participants with severe deficient vitamin D (<25.00 nmoL/L) was younger. Severe deficient vitamin D (<25.00 nmoL/L) was more likely to occur in participants with of female, Non-Hispanic Black and Mexican American, with less physical activity, with obesity or history of CVD and diabetes. In addition, participants with severe deficient vitamin D were less likely to have higher PIR (>300%), have history of cancer, and have mild alcohol consumption.

**Table 1:** Baseline characteristics of long-term prescription opioid users in NHANES, 2001–2018.

Characteristics	Serum 25(OH)D (nmol/l)					
	Total	<25.00	25.00 to < 50.00	50.00 to < 75.00	≥75.00	*p*-value
NO. (%)	1856	101 (5.4)	502 (27.0)	644 (34.7)	609 (32.8)	
Age, mean (SE), y	54.12 (0.5)	53.73 (1.6)	51.76 (0.7)	52.87 (0.7)	56.59 (0.8)	< 0.001
Cotinine, mean (SE), ng/ml*	95.34 (5.5)	105.40 (16.2)	117.04 (11.6)	94.04 (8.4)	83.70 (7.6)	0.07
PA, mean (SE), MET/week	2050.47 (147.2)	1246.69 (410.0)	1433.13 (147.9)	2033.91 (202.5)	2473.86 (293.6)	0.003
Sex, male, *n* (%)	808 (40.7)	38 (27.5)	216 (38.4)	297 (45.5)	257 (38.8)	0.02
Race/ethnicity, *n* (%)						< 0.001
Non-Hispanic White	1,060 (78.5)	26 (44.2)	204 (63.2)	389 (79.2)	441 (89.1)	
Non-Hispanic Black	382 (9.7)	50 (40.1)	152 (18.6)	107 (7.4)	73 (4.2)	
Mexican American	205 (4.4)	15 (8.7)	79 (8.1)	69 (4.1)	42 (2.3)	
Other	209 (7.5)	10 (7.0)	67 (10.1)	79 (9.3)	53 (4.4)	
PIR, *n* (%)						< 0.001
≤130%	809 (30.2)	42 (34.9)	252 (40.2)	291 (31.9)	224 (22.7)	
130–300%	569 (31.0)	40 (44.1)	138 (29.0)	189 (30.1)	202 (31.7)	
>300%	478 (38.9)	19 (21.0)	112 (30.8)	164 (38.0)	183 (45.6)	
Education, *n* (%)						0.13
Less than high school	186 (5.1)	11 (6.5)	55 (6.3)	69 (5.8)	51 (3.6)	
High school	824 (42.0)	45 (40.1)	241 (46.7)	290 (41.8)	248 (39.7)	
More than high school	846 (53.0)	45 (53.4)	206 (47.0)	285 (52.4)	310 (56.7)	
BMI, kg/m^2^, *n* (%)						< 0.001
< 25	396 (22.5)	19 (13.6)	83 (17.6)	135 (22.6)	159 (26.0)	
25–30	540 (31.1)	17 (14.5)	124 (25.3)	196 (31.0)	203 (35.8)	
≥ 30	920 (46.4)	65 (71.9)	295 (57.1)	313 (46.4)	247 (38.2)	
Alcohol consumption, *n* (%)						0.004
Never	203 (7.6)	19 (15.6)	58 (9.5)	61 (5.8)	65 (7.5)	
Former	557 (26.7)	30 (32.9)	159 (29.9)	194 (27.5)	174 (23.6)	
Mild	557 (32.9)	19 (17.6)	150 (29.9)	194 (32.4)	194 (36.3)	
Moderate	246 (17.0)	14 (14.9)	52 (12.4)	89 (17.0)	91 (19.8)	
Heavy	293 (15.8)	19 (19.0)	83 (18.4)	106 (17.4)	85 (12.7)	
Hypertension, *n* (%)	1,352 (68.3)	77 (76.3)	367 (70.1)	455 (65.5)	453 (69.1)	0.30
CVD, *n* (%)	467 (19.7)	34 (36.9)	139 (25.0)	143 (16.3)	151 (18.5)	< 0.001
Diabetes, *n* (%)	526 (21.6)	47 (46.7)	162 (27.0)	163 (19.6)	154 (18.3)	< 0.001
Cancer, *n* (%)	320 (18.4)	12 (12.3)	63 (15.4)	106 (16.5)	139 (22.2)	0.03

NHANES, National Health and Nutrition Examination Survey; SE, standard error; MET, metabolic equivalent; PIR, poverty-income ratio; PA, physical activity; BMI, body mass index; CVD, cardiovascular disease; 25(OH)D, 25-hydroxyvitamin D.

^*^SI conversion factor: to convert cotinine to nanomoles per liter, multiply by 5.675.

All estimates accounted for survey weights; *p*-values for continuous variables and categorical variables were calculated with one-way ANOVA and chi-square test, respectively.

During a median follow-up of 7.75 years, we identified 443 all-cause deaths (weighted, 18.7%), including 135 CVD deaths (weighted, 4.9%), and 94 cancer deaths (weighted, 3.8%).

### 3.2 Associations of serum 25(OH)D concentrations and mortality in long-term prescription opioid users

Table 2 displays the results of Cox proportional hazards regression analyses between serum 25(OH)D concentrations and mortality. After multivariable adjustment (model3), compared to participants with serum 25(OH)D concentrations <25.00 nmol/L, the hazard ratio (HR) for all-cause mortality for those with serum 25(OH)D concentrations ranging from 50.00 to <75.00 nmol/L was 0.50 (95% confidence interval [CI] 0.29, 0.86), for those with serum 25(OH)D concentrations ≥75 nmol/L was 0.54 (95% CI 0.32, 0.90). Nevertheless, no significant inverse associations were observed for serum 25(OH)D concentrations with CVD-specific mortality and cancer-specific mortality after multivariable adjustment. It is important to note that there was no linear association between serum 25(OH)D concentrations and all-cause mortality (*p* for trend = 0.19).

**Table 2:** HRs (95% CI) for mortality risk according to serum 25(OH)D concentrations in long-term prescription opioid users in NHANES, 2001–2018.

	Serum 25(OH)D (nmol/l)				*p* for trend
	<25.00	25.00 to < 50.00	50.00 to < 75.00	≥75.00	
All-cause mortality					
No. of deaths (%)	36 (32.9%)	123 (22.1%)	160 (17.7%)	124 (16.4%)	
Model 1	1.00 (reference)	0.54 (0.33, 0.90)	0.47 (0.30, 0.74)	0.56 (0.36, 0.88)	0.38
Model 2	1.00 (reference)	0.54 (0.33, 0.88)	0.41 (0.25, 0.66)	0.42 (0.27, 0.67)	0.02
Model 3	1.00 (reference)	0.58 (0.34, 1.01)	0.50 (0.29, 0.86)	0.54 (0.32, 0.90)	0.19
CVD-specific mortality					
No. of deaths (%)	16 (13.5%)	36 (5.9%)	48 (4.2%)	35 (4.3%)	
Model 1	1.00 (reference)	0.34 (0.16, 0.71)	0.26 (0.12, 0.56)	0.33 (0.15, 0.72)	0.22
Model 2	1.00 (reference)	0.41 (0.19, 0.89)	0.30 (0.13, 0.66)	0.28 (0.12, 0.66)	0.02
Model 3	1.00 (reference)	0.57 (0.26, 1.24)	0.49 (0.21, 1.12)	0.49 (0.21, 1.12)	0.19
Cancer-specific mortality					
No. of deaths (%)	4 (4.3%)	25 (4.0%)	40 (4.9%)	25 (2.8%)	
Model 1	1.00 (reference)	0.73 (0.19, 2.76)	0.92 (0.22, 3.81)	0.67 (0.17, 2.56)	0.66
Model 2	1.00 (reference)	0.79 (0.18, 3.38)	0.78 (0.16, 3.79)	0.45 (0.10, 2.01)	0.10
Model 3	1.00 (reference)	0.68 (0.13, 3.60)	0.75 (0.12, 4.92)	0.46 (0.08, 2.65)	0.21

SI conversion factor: to convert cotinine to nanomoles per liter, multiply by 5.675.

Model 1: unadjusted. Model 2: adjusted for age, sex, race/ethnicity. Model 3: further adjusted for education, alcohol consumption, cotinine, physical activity, BMI, PIR, hypertension, diabetes, cancer, CVD.

NHANES, National Health and Nutrition Examination Survey; HR, hazard ratio; CI, confidence interval; PIR, poverty-income ratio; BMI, body mass index; CVD, cardiovascular disease; 25(OH)D, 25-hydroxyvitamin D.

### 3.3 Dose–response relationship of serum 25(OH)D concentrations and all-cause mortality in long-term prescription opioid users

**Figure 1:**
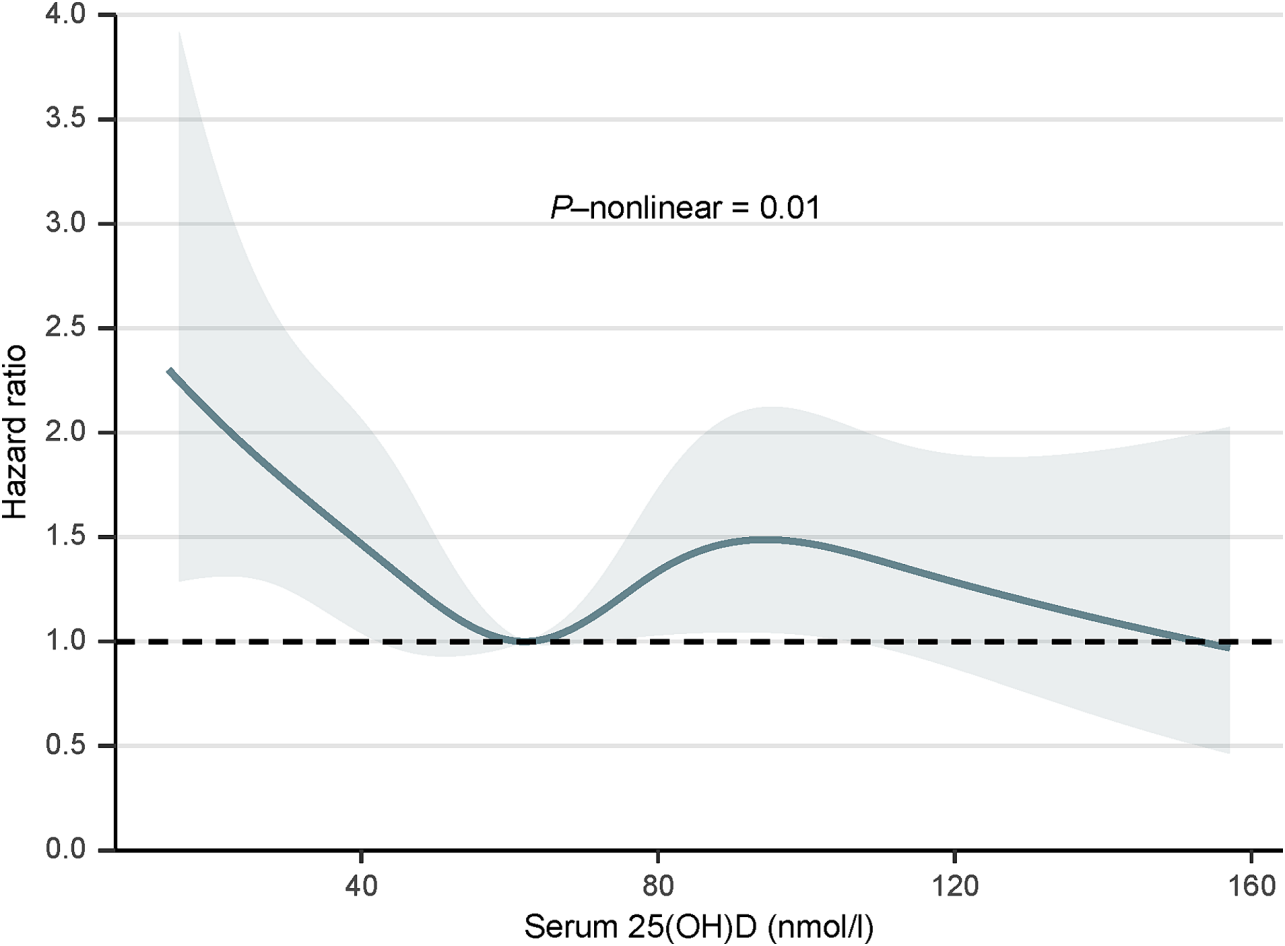
Dose–response relationship between serum 25(OH)D concentrations and all-cause mortality, 2001 to 2018. The model was adjusted for age, sex, race/ethnicity, education, alcohol consumption, cotinine, physical activity, BMI, PIR, hypertension, diabetes, cancer, and CVD. SI conversion factor: to convert cotinine to nanomoles per liter, multiply by 5.675. PIR, poverty-income ratio; BMI, body mass index; CVD, cardiovascular disease; 25(OH)D, 25-hydroxyvitamin D.

We used the RCS curve to estimate the dose–response relationship between serum 25(OH)D concentrations and all-cause mortality, and a non-linear association was observed after adjusting all confounders (*p* for non-linear = 0.01) (Figure 1). The inflection points for serum 25(OH)D concentrations were 62.17 nmoL/L and 94.52 nmol/L. Subsequently, piecewise COX proportional hazards regression was performed to analyze a threshold effect of serum 25(OH)D concentrations on all-cause mortality (Table 3). When serum 25(OH)D concentrations were < 62.17 nmoL/L, per 1-unit increment in serum 25(OH)D concentrations, there was a 2% lower risk of all-cause mortality (HR 0.98, 95%CI [0.97, 1.00]). However, there was no benefit effect for lower risk of all-cause mortality when elevate serum 25(OH)D concentrations for participants with serum 25(OH)D concentrations were 62.17- <94.52 nmol/L (HR 1.01, 95%CI [0.98, 1.03]) or ≥ 94.52 nmol/L (HR 0.97, 95%CI [0.93, 1.02]).

**Table 3:** Threshold effect analysis of serum 25(OH)D concentrations on all-cause mortality in long-term prescription opioid users in NHANES, 2001–2018.

All-cause mortality	Adjusted HR (95% CI), *p*-value
Inflection point	62.17 nmoL/L, 94.52 nmol/L
Per 1-unit increment in 25(OH)D concentrations <62.17 nmol/L	0.98 (0.97, 1.00), 0.02
Per 1-unit increment in 25(OH)D concentrations 62.17- < 94.52 nmol/L	1.01 (0.98,1.03), 0.57
Per 1-unit increment in 25(OH)D concentrations ≥94.52 nmol/L	0.97 (0.93,1.02), 0.22

Adjusted for age, sex, race/ethnicity, education, alcohol consumption, cotinine, physical activity, BMI, PIR, hypertension, diabetes, cancer, CVD.

NHANES, National Health and Nutrition Examination Survey; HR, hazard ratio; CI, confidence interval; PIR, poverty-income ratio; BMI, body mass index; CVD, cardiovascular disease; 25(OH)D, 25-hydroxyvitamin D.

### 3.4 Stratified and sensitivity analyses

**Figure 2:**
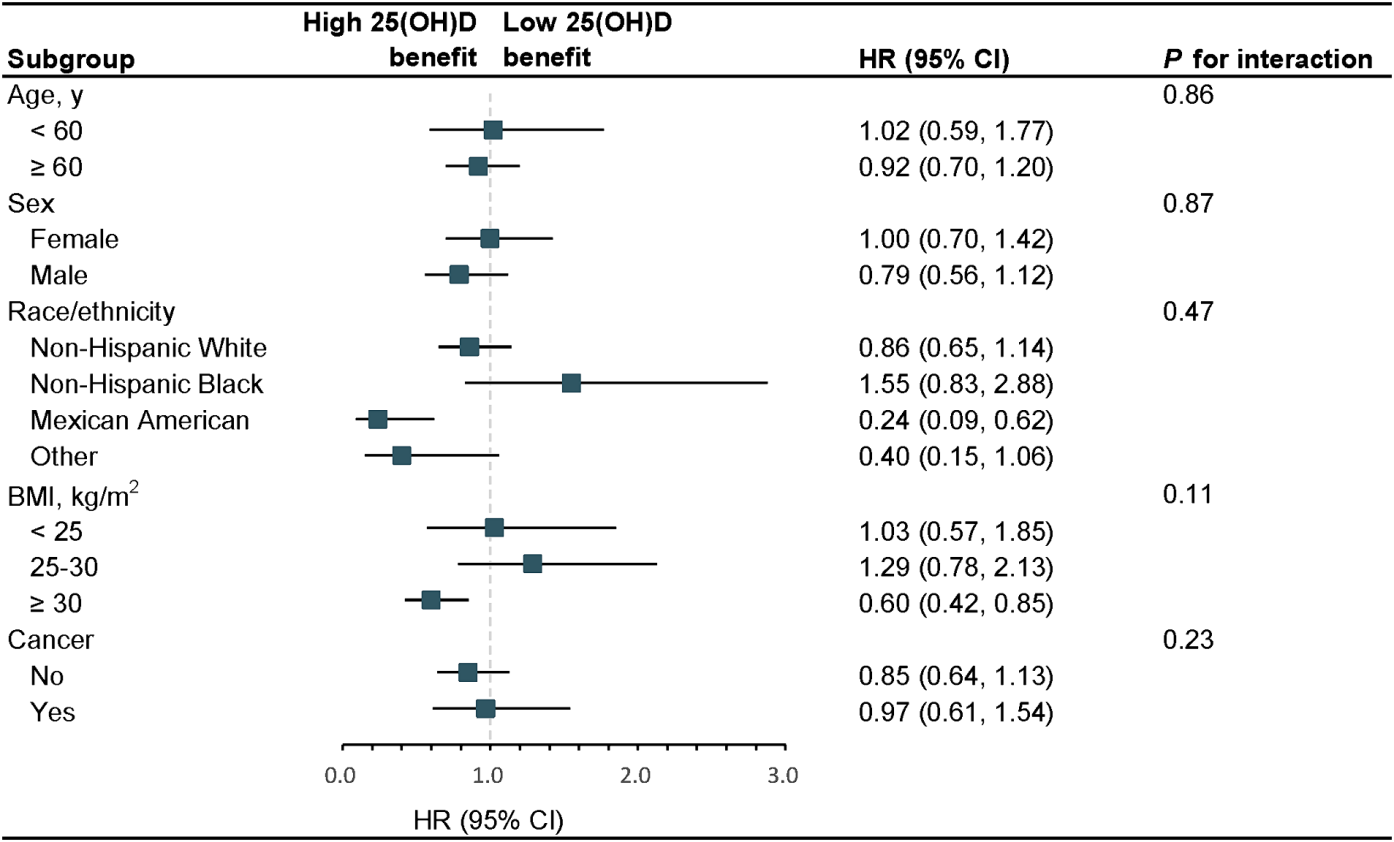
Stratified analyses of serum 25(OH)D concentrations with the risk of all-cause mortality, 2001–2018. The model was adjusted for age, sex, race/ethnicity, education, alcohol consumption, cotinine, physical activity, BMI, PIR, hypertension, diabetes, cancer, and CVD. The strata variable was not included in the adjustment when stratifying by itself. SI conversion factor: to convert cotinine to nanomoles per liter, multiply by 5.675. HR, hazard ratio; CI, confidence interval; PIR, poverty-income ratio; BMI, body mass index; CVD, cardiovascular disease; 25(OH)D, 25-hydroxyvitamin D.

In stratified analyses, the benefit of high serum 25(OH)D concentrations (≥ 62.17 nmol/L) and low serum 25(OH)D concentrations (< 62.17 nmol/L) for the survival rate of participants with long-term prescription opioid use stratified by age, sex, race/ethnicity, BMI, and cancer was similar. No modification factors were found between serum 25(OH)D concentrations and these stratified variables (all *p* for interaction >0.05) (Figure 2). In sensitivity analyses, the results were also typically robust when excluding deaths that occurred within 2 years (Supplementary Table S1); excluding participants with history of CVD or cancer (Supplementary Table S2); further adjustment of blood drawing season (Supplementary Table S3).

## 4 Discussion

In this prospective cohort study of participants with long-term prescription opioid use, we discovered significantly non-linear inverse association between serum 25(OH)D concentrations and all-cause mortality, nevertheless, no inverse associations were observed for serum 25(OH)D concentrations with CVD-specific mortality and cancer-specific mortality. Lower vitamin D in participants with serum 25(OH)D concentrations < 62.17 nmol/L were associated with an increased risk of all-cause mortality. Stratified and sensitivity analyses supported the stability of our conclusions.

Our research revealed that a significant proportion (67.2%) of long-term prescription opioid users had insufficient serum 25(OH)D concentrations, highlighting a prevalent vitamin D deficiency within this population. Preclinical study have demonstrated that vitamin D deficiency accelerated the development of opioid tolerance and exacerbated opioid dependence, resulting in elevated opioid consumption (16). Turner et al. (15) have reported that chronic pain patients with vitamin D deficiency exhibited significantly greater prescription opioid doses and prolonged duration of use. Excessive or long-term use of prescription opioids undoubtedly increases the risk of mortality. Consequently, there is a basis to hypothesize that vitamin D deficiency heightens the mortality risk in long-term prescription opioid users. Prior epidemiological studies have indicated the association between low serum 25(OH)D concentrations and increased all-cause mortality risk within the general population or specific subgroups (27–31), aligning with our findings. The majority of studies have demonstrated that low serum 25(OH)D concentrations are associated with increased incidence of cardiovascular events and cardiovascular mortality (32–34). Nevertheless, our study did not observe a relationship between low serum 25(OH)D concentrations and the risk of cardiovascular mortality, potentially attributable to the focus on specific high-risk population, a smaller sample size, and differences in ethnicity. Likewise, we did not detect low serum 25(OH)D concentrations linked to an increased risk of cancer mortality. The association between vitamin D status and cancer mortality was controversial (35). A meta-analysis conducted by Chowdhury et al. (36) proposed that elevated baseline serum 25(OH)D concentrations were linked to a decreased risk of cancer mortality. Conversely, several other extensive prospective studies failed to identify an association between serum 25(OH)D concentrations and the risk of cancer mortality (37–39).

In long-term prescription opioid users, we observed a non-linear relationship between serum 25(OH)D concentrations and the risk of all-cause mortality (27, 40, 41), aligning with earlier findings in diverse populations. In comparison to the optimal serum 25(OH)D concentrations reported in other populations, our study revealed that sustaining serum 25(OH)D concentrations above 62.17 nmol/L corresponded to a decreased risk of all-cause mortality in long-term prescription opioid users. Collectively, these studies indicate the presence of a ceiling effect for serum 25(OH)D concentrations concerning health outcomes, including mortality, wherein surpassing a specific threshold may not yield additional advantages. Although consensus regarding the ideal serum 25(OH)D concentrations is lacking, the National Institutes of Health advised maintaining levels above 50.00 ng/mL to mitigate the health hazards linked to vitamin D insufficiency (42). Despite observational studies suggest that low serum 25(OH)D concentrations contribute to mortality risk, recent intervention studies have failed to demonstrate the advantages of vitamin D supplementation (12–14). Likewise, Neale et al. (43) observed that monthly vitamin D supplementation in the elderly did not result in lower all-cause mortality. A meta-analysis conducted by Zhang et al. (44) indicated that vitamin D supplementation alone only reduced cancer mortality in the general population. Wu et al. (45) discovered that monthly vitamin D supplementation did not lead to a reduction in prescription opioid use. However, these experiments included participants with high baseline serum 25(OH)D concentrations, such as 115 nmol/L in Neale RE et al.’s study and 66.4 nmol/L in Wu et al.’s study (exceeding 50 nmol/L), thereby constraining the efficacy of supplementation tests within the low serum 25(OH)D concentration subgroups. Subsequent studies exploring the potential of vitamin D supplements to mitigate all-cause mortality in long-term prescription opioid users should consider enrolling participants with lower baseline serum 25(OH)D concentrations.

This study possesses several strengths. Firstly, to the best of our knowledge, this was the first study to investigate the relationship between vitamin D deficiency and mortality in long-term prescription opioid users. Secondly, this study relied on a nationally representative sample and exhibited excellent follow-up rates. Lastly, several sensitivity analyses were performed to affirm the model’s stability, thereby bolstering the reliability of our study’s findings. Nonetheless, this study possesses certain limitations. Firstly, owing to its nature of cross-sectional design, it was unable to establish a causal relationship between vitamin D deficiency and mortality. Then, despite numerous potential confounding factors had been adjusted for, residual confounding factors might persist. Additionally, the absence of repeated measurements of serum 25(OH)D concentrations precluded the evaluation of the influence of dynamic fluctuations in vitamin D status on mortality.

## 5 Conclusion

In conclusion, a non-linear association was observed between serum 25(OH)D concentrations and all-cause mortality in long-term prescription opioid users. Lower vitamin D in participants with serum 25(OH)D concentrations < 62.17 nmol/L were associated with an increased risk of all-cause mortality.

## Data availability statement

Publicly available datasets were analyzed in this study. This data can be found at: http://www.cdc.gov/nchs/nhanes.htm.

## Ethics statement

The studies involving humans were approved by NCHS Ethics Review Board, National Center for Health Services. The studies were conducted in accordance with the local legislation and institutional requirements. The participants provided their written informed consent to participate in this study.

## Author contributions

SD: Conceptualization, Formal analysis, Investigation, Methodology, Writing – original draft, Writing – review & editing. JW: Data curation, Investigation, Writing – review & editing. PW: Data curation, Validation, Visualization, Writing – review & editing. ZH: Conceptualization, Project administration, Supervision, Writing – review & editing.
