# Supplementary material for: Associations of vitamin D status with all-cause and cause-specific mortality in long-term prescription opioid users
Source: Front Nutr. 2024 Jun 18;11:1422084. doi: 10.3389/fnut.2024.1422084 (PMC11217488; doi:10.3389/fnut.2024.1422084)
Supplement: Supplementary file 1 [file Data_Sheet_1.docx]

**Figure S1. Flowchart of the study participants in NHANES, 2001 to 2018**

**91,351** Participants in 2001-2018 NHANES

**1856** Final participants with complete data

**50,201** Adults aged ≥ 20 y

**41,150** Excluded for age < 20 y

**47,426** Excluded for non-long-term prescription opioid user

**79** Excluded for receiving medications for opioid dependence or withdrawal

**331** Excluded for missing value of serum 25(OH)D concentrations

**1** Excluded for unavailable value of follow-up

**508** Excluded for missing value of covariates

**Table S1.** **HRs (95% CI) for mortality risk according to serum 25(OH)D concentrations for participants after excluding deaths occurred within 2 years in NHANES, 2001 to 2018**

|  | **Serum 25(OH)D (nmol/l)** | | | | ***P* for trend** |
| --- | --- | --- | --- | --- | --- |
|  | **<25.00** | **25.00 to <50.00** | **50.00 to <75.00** | **≥75.00** |  |
| **All-cause mortality** |  |  |  |  |  |
| No. of deaths (%) | 32 (30.7%) | 105 (20.3%) | 131 (15.4%) | 101 (13.8%) |  |
| Model 1 | 1.00 (reference) | 0.52 (0.30, 0.90) | 0.43 (0.26, 0.71) | 0.51 (0.32, 0.84) | 0.22 |
| Model 2 | 1.00 (reference) | 0.50 (0.28, 0.90) | 0.37 (0.21, 0.64) | 0.39 (0.23, 0.66) | 0.01 |
| Model 3 | 1.00 (reference) | 0.55 (0.30, 1.02) | **0.45 (0.25, 0.83)** | **0.50 (0.28, 0.88)** | 0.16 |
| **CVD-specific mortality** |  |  |  |  |  |
| No. of deaths (%) | 15 (12.7%) | 31 (5.6%) | 36 (3.3%) | 29 (3.2%) |  |
| Model 1 | 1.00 (reference) | 0.33 (0.15, 0.72) | 0.21 (0.10, 0.47) | 0.27 (0.12, 0.61) | 0.06 |
| Model 2 | 1.00 (reference) | 0.41 (0.17, 0.95) | 0.27 (0.11, 0.65) | 0.26 (0.10, 0.67) | 0.01 |
| Model 3 | 1.00 (reference) | 0.60 (0.25, 1.47) | 0.45 (0.19, 1.07) | 0.47 (0.18, 1.25) | 0.14 |
| **Cancer-specific mortality** |  |  |  |  |  |
| No. of deaths (%) | 2 (2.9%) | 19 (3.2%) | 33 (4.2%) | 20 (2.4%) |  |
| Model 1 | 1.00 (reference) | 0.85 (0.14, 5.01) | 1.18 (0.17, 8.12) | 0.89 (0.15, 5.38) | 0.98 |
| Model 2 | 1.00 (reference) | 0.95 (0.15, 6.26) | 1.11 (0.14, 8.67) | 0.68 (0.10, 4.66) | 0.34 |
| Model 3 | 1.00 (reference) | 0.74 (0.10, 5.72) | 0.97 (0.10, 9.71) | 0.61 (0.07, 5.19) | 0.49 |

SI conversion factor: To convert cotinine to nanomoles per liter, multiply by 5.675.

Model 1: unadjusted. Model 2: adjusted for age, sex, race/ethnicity. Model 3: further adjusted for education, alcohol consumption, cotinine, physical activity, BMI, PIR, hypertension, diabetes, cancer, CVD.

Abbreviations: NHANES, National Health and Nutrition Examination Survey; HR, hazard ratio; CI, confidence interval; PIR, poverty-income ratio; BMI, body mass index; CVD, cardiovascular disease; 25(OH)D, 25-hydroxyvitamin D.

**Table S2.** **HRs (95% CI) for mortality risk according to serum 25(OH)D concentrations for participants without history of CVD or cancer in NHANES, 2001 to 2018**

|  | **Serum 25(OH)D (nmol/l)** | | | | ***P* for trend** |
| --- | --- | --- | --- | --- | --- |
|  | **<25.00** | **25.00 to <50.00** | **50.00 to <75.00** | **≥75.00** |  |
| **All-cause mortality** |  |  |  |  |  |
| No. of deaths (%) | 15 (22.8%) | 56 (15.6%) | 80 (13.8%) | 42 (9.6%) |  |
| Model 1 | 1.00 (reference) | 0.56 (0.30,1.06) | 0.52 (0.29, 0.95) | 0.47 (0.24, 0.92) | 0.21 |
| Model 2 | 1.00 (reference) | 0.49 (0.24, 1.01) | 0.42 (0.21, 0.86) | 0.34 (0.16, 0.75) | 0.05 |
| Model 3 | 1.00 (reference) | 0.55 (0.27, 1.12) | **0.45 (0.22, 0.90)** | **0.38 (0.17, 0.84)** | 0.08 |
| **CVD-specific mortality** |  |  |  |  |  |
| No. of deaths (%) | 4 (4.9%) | 16 (3.2%) | 23 (3.3%) | 13 (3.0%) |  |
| Model 1 | 1.00 (reference) | 0.45 (0.11, 1.92) | 0.50 (0.15, 1.71) | 0.56 (0.14, 2.34) | 0.94 |
| Model 2 | 1.00 (reference) | 0.45 (0.12, 1.72) | 0.59 (0.16, 2.23) | 0.52 (0.12, 2.21) | 0.91 |
| Model 3 | 1.00 (reference) | 0.58 (0.13, 2.60) | 0.68 (0.16, 3.01) | 0.63 (0.11, 3.58) | 0.89 |
| **Cancer-specific mortality** |  |  |  |  |  |
| No. of deaths (%) | 2 (4.6%) | 9 (2.2%) | 13 (2.5%) | 5 (0.7%) |  |
| Model 1 | 1.00 (reference) | 0.37 (0.06, 2.30) | 0.45 (0.08, 2.60) | 0.15 (0.02,1.05) | 0.08 |
| Model 2 | 1.00 (reference) | 0.38 (0.05, 3.08) | 0.38 (0.04, 3.24) | 0.10 (0.01,1.04) | 0.02 |
| Model 3 | 1.00 (reference) | 0.38 (0.04, 4.01) | 0.30 (0.02, 3.56) | 0.09 (0.01, 1.17) | 0.02 |

SI conversion factor: To convert cotinine to nanomoles per liter, multiply by 5.675.

Model 1: unadjusted. Model 2: adjusted for age, sex, race/ethnicity. Model 3: further adjusted for education, alcohol consumption, cotinine, physical activity, BMI, PIR, hypertension, diabetes, cancer, CVD.

Abbreviations: NHANES, National Health and Nutrition Examination Survey; HR, hazard ratio; CI, confidence interval; PIR, poverty-income ratio; BMI, body mass index; CVD, cardiovascular disease; 25(OH)D, 25-hydroxyvitamin D

**Table S3.** **HRs (95% CI) for mortality risk according to serum 25(OH)D** **concentrations for participants when further adjusted blood drawing season in NHANES, 2001 to 2018**

|  | **Serum 25(OH)D (nmol/l)** | | | | ***P* for trend** |
| --- | --- | --- | --- | --- | --- |
|  | **<25.00** | **25.00 to <50.00** | **50.00 to <75.00** | **≥75.00** |  |
| **All-cause mortality** | 1.00 (reference) | 0.58 (0.33,1.01) | **0.50 (0.29, 0.86)** | **0.54 (0.32, 0.90)** | 0.21 |
| **CVD-specific mortality** | 1.00 (reference) | 0.54 (0.24, 1.19) | 0.49 (0.21, 1.12) | 0.49 (0.21, 1.12) | 0.24 |
| **Cancer-specific mortality** | 1.00 (reference) | 0.66 (0.13, 3.49) | 0.75 (0.12, 4.84) | 0.46 (0.08, 2.62) | 0.22 |

SI conversion factor: To convert cotinine to nanomoles per liter, multiply by 5.675.

The model was adjusted for age, sex, race/ethnicity, education, alcohol consumption, cotinine, physical activity, BMI, PIR, hypertension, diabetes, cancer, CVD, and blood drawing season.

Abbreviations: NHANES, National Health and Nutrition Examination Survey; HR, hazard ratio; CI, confidence interval; PIR, poverty-income ratio; BMI, body mass index; CVD, cardiovascular disease; 25(OH)D, 25-hydroxyvitamin D.
